# Natural Food Colorant Obtained from Wild *Berberis vulgaris* L. by Ultrasound-Assisted Extraction: Optimization and Characterization

**DOI:** 10.3390/foods14020183

**Published:** 2025-01-09

**Authors:** Erika N. Vega, Lorena González-Zamorano, Elena Cebadera, Lillian Barros, Tânia C. S. P. Pires, Adriana K. Molina, Tayse F. F. da Silveira, Guillermo Vidal-Diez de Ulzurrun, Javier Tardío, Montaña Cámara, Virginia Fernández-Ruiz, Patricia Morales

**Affiliations:** 1 Departamento Nutrición y Ciencia de los Alimentos, Facultad de Farmacia, Universidad Complutense de Madrid, Plaza Ramón y Cajal, s/n, 28040 Madrid, Spain; erinino@ucm.es (E.N.V.); lorego06@ucm.es (L.G.-Z.); ecebadera@farm.ucm.es (E.C.); mcamara@ucm.es (M.C.); vfernand@ucm.es (V.F.-R.); 2 Centro de Investigação de Montanha (CIMO), LA SusTEC, Instituto Politécnico de Bragança, Campus de Santa Apolónia, 5300-253 Bragança, Portugal; lillian@ipb.pt (L.B.); tania.pires@ipb.pt (T.C.S.P.P.); amolina@ipb.pt (A.K.M.); tayse.silveira@ipb.pt (T.F.F.d.S.); 3 Institute of Molecular Biology, Academia Sinica, Nangang, 128 Academia Road, Section 2, Taipei 115, Taiwan; gvidal@gate.sinica.edu.tw; 4 Instituto Madrileño de Investigación y Desarrollo Rural, Agrario y Alimentario (IMIDRA), Finca “El Encín”, Apdo. 127, 28800 Alcalá de Henares, Spain; javier.tardio@madrid.org

**Keywords:** food additives, natural food colorant, wild fruits, bioactive compounds, anthocyanins

## Abstract

In this study, a novel natural food colorant based on anthocyanins was developed from wild barberry (*Berberis vulgaris* L.) fruits using ultrasound-assisted extraction, which was optimized through RSM. Four extraction variables (ultrasound power, time, S/L ratio, and extraction solvent pH) were evaluated in combination. The response criteria used were the total anthocyanin content (TAC) and color parameters. The optimal TAC was achieved at 2.5 min, 345 W, pH 3, and 22.12 g/L. The fruit sample (without seeds) (BVFF) and its optimized extract (BVE) were characterized in terms of chemical composition and bioactivities. Delphinidin-3-*O*-glucoside was identified as the predominant anthocyanin. BVE exhibited a total phenolic content of 290.72 mg/g. Additionally, both BVFF and BVE presented significant antioxidant, antibacterial, and antifungal activity, especially in the case of BVE, which inhibited the growth of several foodborne bacteria and fungi and even showed bactericidal capacity against most of the tested bacteria, particularly against *E. cloacae*, *E. coli*, *P. aeruginosa*, and *B. cereus*. These results highlight the richness of BVFF and BVE in bioactive compounds, especially anthocyanins, underscoring their potential as natural food colorants that can be used in food product formulations instead of synthetic azo colorants.

## 1. Introduction

Color is one of the first parameters in contact with the consumer, so it is one of the most influential factors in consumer food choices, providing an idea of the freshness, taste, or composition of food products, which can lead to their acceptability or rejection [1]. To meet consumers’ expectations, the food industry has been using colorants to restore, enhance, or add color to different products [2,3]. Colorants can be classified, according to their source, as artificial colorants, which are obtained synthetically, and natural colorants, which are obtained from vegetables, animals, or minerals. Artificial colorants are the most widely used by the food industry; however, several of them have been related with adverse effects such as urticaria, allergies, alteration of kidney and liver function, and attention deficit and hyperactivity disorder (ADHD), especially in children [4,5,6].

Common food pigments found in vegetables and fruits principally include anthocyanins, betalains, carotenoids, and chlorophylls, with anthocyanins being the most abundant and, therefore, the most studied. To date, more than 700 different anthocyanins have been identified [7].

*Berberis vulgaris* L. (Berberidaceae) is a spiny shrub native to Asia, Europe, Africa, North America, and South America, but Iran is currently the largest producer [7]. Its blackish-red and sour-tasting fruits have been traditionally consumed in the form of sauce, jelly, marmalade, and jam [7,8,9]. In addition, there are some reports of the traditional medicinal use of *B. vulgaris* as far back as 650 BC, mainly for the treatment of eye, ear, and mouth infections, as well as for treating hemorrhoids, kidney stones, and indigestion [7,9,10].

One of the most studied bioactive compounds in *B. vulgaris* is berberine, an isoquinoline alkaloid that presents several bioactivities such as antioxidant, antimicrobial, anti-inflammatory, and antidiabetic properties, among others. However, in 2019, the French Agency for Food, Environmental and Occupational Health & Safety (ANSES) requested that the EFSA provided a scientific opinion on supplements containing berberine after evidence emerged suggesting that these supplements may cause adverse effects including gastrointestinal disorders, hypoglycemia, and hypotension, an evaluation that is currently ongoing [11]. Moreover, berberine is predominantly concentrated in the roots and stems of this plant [12]. The fruits of *B. vulgaris* contain dextrose and fructose, organic acids like tartaric acid and citric acid, and several vitamins, especially vitamins C and provitamin A, as well as essential minerals such as calcium, iron, and potassium. Moreover, the fruit is a remarkable source of anthocyanins, which serve as its primary pigment [7,13].

The growing interest in natural food colorant development has led to the exploration of advanced extraction methodologies, with ultrasound-assisted extraction (UAE) emerging as a particularly efficient method. More specifically, UAE has shown higher efficiency in the extraction of anthocyanins from several matrices compared to other extractions methods, such as maceration or microwave-assisted extraction (MAE). For instance, maceration requires the use of high temperatures that can degrade anthocyanins, as well as significant amounts of solvents [14]. Similarly, MAE induces the migration of dissolved molecules but it has been associated with greater thermal degradation of bioactive molecules [14,15].

The excellent results obtained with the UAE methodology are attributed to its working mechanism: cavitation. In this process, ultrasound radiation causes the rupture of plant tissue, allowing for better penetration of solvents and resulting in faster extraction times, reducing solvent use and, thus, supporting the principles of green chemistry [16]. However, since each matrix has a unique anthocyanin profile, it is essential to optimize the extraction conditions for each kind of fruit. One of the most widely used methodologies is the response surface methodology (RSM), which predicts the optimal experimental conditions by analyzing the effects of multiple independent variables [17].

Nowadays, human beings are more aware of the importance and influence of food on their health, which has increased the demand for healthier food products, made without additives or with natural ones [18]. This fact has led to increasing research on the development of colorants from natural sources, which, unlike artificial ones, not only lack harmful side effects but also present diverse bioactive properties with potential beneficial effects on consumers’ health.

According to the above, the main objective of the present study was to determine the optimal conditions for the extraction of anthocyanins from *Berberis vulgaris* fruits through UAE, focusing on factors such as the liquid/solid ratio, ultrasound power, solvent pH, and extraction time through the use of RSM. Additionally, in this study, both the fruits and the optimized developed extract were characterized in terms of anthocyanins, bioactive compounds, and bioactive properties. The extraction conditions and chemical composition of the *B. vulgaris* extract have been previously filed to the Spanish Patent Office (SPO) as part of patent ES-2990137 A1.

## 2. Materials and Methods

### 2.1. Samples

Mature fruits of wild *Berberis vulgaris* L. (BVF) were collected from two different Spanish locations during two consecutive years (2022 and 2023) (Table 1), in order to obtain a representative sample that reflects the possible natural variability of this fruit’s endemic collection areas, with harvesting permission given under Ref. PN-NC_022022 and Ref. ABSCH-IRCC-ES-262067-1 issued by the Spanish Ministry of Agriculture, Fisheries and Food (Ministerio de Agricultura, Pesca y Alimentación, MAPA). After physical–chemical characterization, the whole fruits (BVF, *Berberis vulgaris* fruit) were frozen and lyophilized at −80 °C (±5 °C) and 0.029 mbar (Freezone; 4.5 L; LABCONCO), and the seeds were then removed as a by-product. The pulp and skin fractions from all gathered samples (from both locations and harvesting years) were milled together using an IKA Multidrive Basic (BS000) in order to obtain a homogenized and representative sample (named BVFF, with the *Berberis vulgaris* fruit fraction as pool sample) used in further analysis.

Moreover, after the optimization extraction process, *Berberis vulgaris* extract (BVE) was obtained and used for further analysis. All samples were stored at −20 °C in the absence of light until analysis to preserve the stability of anthocyanins, which are highly sensitive to external factors such as light or heat.

The analytical methods were performed in the following *Berberis vulgaris* samples:

(1) Fresh fruit sample (BVF, *Berberis vulgaris* fruit): whole fruit with seeds used for the initial physicochemical analysis.

(2) Freeze-dried fruit fraction sample (BVFF, *Berberis vulgaris* fruit fraction): fruits without seeds used for determining the optimal extraction conditions by ultrasound-assisted extraction, as well as for developing an optimized colorant extract (BVE, *Berberis vulgaris* extract).

(3) BVFF and BVE: used for chemical analysis, namely anthocyanin characterization, total polyphenol and phenolic family determination, and bioactive property analysis (antioxidant and antimicrobial assays).

### 2.2. Determination of the Optimal Extraction Conditions Through RSM

#### 2.2.1. Experimental Design

Based on preliminary studies four factors were selected as the experimental variables to evaluate their combined influence on anthocyanin extraction, namely solvent pH, ultrasound power (W), extraction time (min), and S/L ratio (mg/mL). Time was evaluated at five levels from 2.5 to 20 min, ultrasound power at three levels from 250 to 500 W, while solid/liquid ratio and solvent pH were evaluated at two levels: 16.33–33.33 g/mL and 3–6, respectively. All the tests were carried out in duplicate; therefore, a total of 120 assays were performed as a result of the 60 different variable combinations (Table 2).

#### 2.2.2. Ultrasound-Assisted Extraction Process

For the ultrasound extraction process, the *Berberis vulgaris* freeze-dried fruit fraction (BVFF) sample was used, where BVFF corresponds to the combined freeze-dried fruit fraction samples from the two different locations. As mentioned in the Materials and Methods section, this approach allows for the generation of a homogenous and significant sample, facilitating the development of an optimized ultrasound-assisted extraction process that can be applied to *B. vulgaris* fruits.

Ultrasound-assisted extraction was applied to 0.25 g of sample using ethanol and water at a ratio of 80:20 (*v*/*v*) as the extraction solvent. Moreover, the temperature of the ultrasound process was controlled at 30 °C. For the extraction process, the sample was combined with the corresponding volume of extraction solvent (16.66 or 33.33 mg/mL) at the corresponding pH (3 or 6), mixed and subjected to the corresponding extraction conditions (time between 2.5 and 20 min and ultrasound power between 250 and 500 W) in a Sonic Dismembrator model 705 (Fisherbrand, Waltham, MA, USA). After the ultrasound process, samples were centrifugated at 3500 rpm for 5 min, and the supernatant was collected. A small portion of the supernatant was used for determination of the total anthocyanin content (see the section titled “Determination of Total Monomeric Anthocyanin Content”) and the color parameters (Section 2.3.2). The remaining supernatant had the ethanol eliminated by rotatory evaporation (Rotavapor R-114; BÜCHI), then the aqueous extract was frozen at −20 °C and freeze-dried at −80 °C and 0.029 mbar (Freezone; 4.5 L; LABCONCO) to obtain a solid extract (optimized colorant extract, BVE).

#### 2.2.3. Response Criteria Used to Evaluate the Extraction Process

For the evaluation and determination of the optimal anthocyanin extraction conditions from *Berberis vulgaris*, the total anthocyanin content (see the section titled “Determination of Total Monomeric Anthocyanin Content”), saturation index (C*), and hue (h) (Section 2.3.2) were used.

#### 2.2.4. Mathematical Model: Response Surface Methodology (RSM)

A response surface methodology (RSM) was developed to investigate the influence of various experimental conditions on anthocyanin content. The variables analyzed included pH (X_1_), ultrasound power used (X_2_), time (X_3_), and solid/liquid ratio (X_4_). The RSM was constructed by fitting experimental data from the 120 assays described in Section 2.2.1 to the following second-order polynomial equation (Equation (1)):

(1) $$Y=b_{0}+\sum_{i=1}^{n}b_{i}X_{i}+\sum_{\begin{array}{c} i=1\\ j>1 \end{array}}^{n-1}\sum_{j=2}^{n}b_{ij}X_{i}X_{j}+\sum_{i=1}^{n}b_{ii}X_{i}^{2}$$

Here, Y denotes the response variable, namely anthocyanin content. The coefficients b_0_, b_i_, b_ij_, and b_ii_ represent the constant, linear, interaction and quadratic effects, respectively, while *n* represents the number of variables (four in this case: solvent pH, ultrasound power (W), extraction time (min), and S/L ratio (mg/mL)). The NonLinearModelFit function from Mathematica (Version 11.1.1.0, Wolfram Research Inc., Champaign, IL, USA) was used to compute the RSM coefficients as well as the coefficient of determination (R^2^) that was used to assess the accuracy of the model. It is important to note that while the developed model functions as a response surface model, the data collection process did not adhere to traditional RSM design due to specific constraints.

The RSM model served to determine the optimal extraction conditions for maximizing anthocyanin content. These values were found using the FindMaximum function (also from Mathematica) with default parameters and the following constrains: 3 ≤ X_1_ ≤ 6, 50 ≤ X_2_ ≤ 100, 2.5 ≤ X_3_ ≤ 20, and 16.6 ≤ X_4_ ≤ 33.3 (as observed in Table 2).

### 2.3. Analytical Determinations

#### 2.3.1. Physical–Chemical Analysis

BVFs from both locations were analyzed in terms of moisture, pH, and titratable acidity. Moisture was determined by weight difference according to the AOAC 984.25 method [19]. pH was measured according to the AOAC 981.12 method [19]. Titratable acidity was analyzed by acid–base volumetry, as described in the AOAC 942.15 method [19]. All determinations were performed in triplicate (n = 3).

#### 2.3.2. Color Characterization: CIELAB Parameters

The color parameters were measured in BVFF and BVE using the tristimulus colorimetry method previously described by Loughrey [20]. In the case of the evaluation of the extraction process, the color was analyzed in 15 mL of the liquid extract for each of the 120 assays. For the characterization of the freeze-dried fruit sample and freeze-dried optimized extract, the solid sample was placed in a cuvette until it fully covered the base. In both cases, color was measured using a ColorFlex colorimeter from Hunterlab under the following parameters: CIE L*a*b* color space, illumination C, 10° and 45/0° geometry. Saturation index (C*) and hue angle (h) were calculated from a* and b* parameters according to the following equations [20,21]:

C*_ab_ = (a*^2^ + b*^2^)^1/2^ (2)

h_ab_ = arctan (b*/a*) (3)

#### 2.3.3. Anthocyanin Characterization: Total Monomeric Anthocyanin and Individual Anthocyanin Profile

##### Determination of Total Monomeric Anthocyanin Content

The determination of the total anthocyanin (TAC) content was evaluated in BVFF and BVE through the pH differential method [22]. For the evaluation of the extraction process, the assay was carried out with 100 µL of the liquid extract. In the case of the characterization of the freeze-dried fruit sample and optimized colorant extract, the assay was carried out with 10 ± 0.1 mg of each sample through the QUENCHER (Quick, Easy, New, CHEap and Reproducible) methodology [23,24]. Briefly, 10 mL of either KCl (Panreac, Barcelona, España) buffer (pH 1) or CH_3_CO_2_Na (Panreac, Barcelona, España) buffer (pH 4.5) was added to the sample, homogenized by vortexing, and placed on an orbital shaker. After 15 min, the sample was centrifugated for 5 min at 7000 rpm and filtered. Finally, the absorbance was measured at 510 and 700 nm in a UV-vis microplate reader (Synergy HTX, Biotek, Santa Clara, CA, USA). A cyanidin-3-*O*-glucoside calibration curve was obtained with concentrations from 3.125 to 500 µg/mL; thus, the results were expressed as milligrams of cyanidin-3-glucoside (Sigma-Aldrich, Schnelldorf, Germany) per gram of sample (mg cya-3-glu/g, dw).

##### Individual Anthocyanin Profile

The individual anthocyanin characterization was evaluated in BVFF and BVE. For the identification of the individual anthocyanins, the dissolved extract was injected into an HPLC system (Dionex Ultimate 3000 UPLC, Thermo Scientific, San Jose, CA, USA) coupled to a diode-array detector monitoring at 520 nm and a mass spectrometer (Linear Ion Trap LTQ XL, Thermo Scientific, San Jose, CA, USA) equipped with an electrospray (ESI) ionization source working in positive mode. For compound separation, an AQUA^®^ reverse-phase C18 column (5 µm, 150 mm × 4.6 mm, Phenomenex, Alcobendas, Spain) was used at 35 °C, using the gradient previously described by Gonçalves et al. [25]. The anthocyanin identification was carried out by comparison of the retention time and UV-vis and mass spectra with authentic standards and literature data.

For the quantification of the individual anthocyanins, the water-dissolved extract (10 mg/mL) was injected into the UHPLC equipment (series 1290 Infinity II, Agilent Technologies, CA, USA) coupled with a diode-array detector (DAD, series 1260 Infinity II, Agilent Technologies, CA, USA) as detailed by Vega et al. [26]. A Poroshell 120 SB-C18 column (4.6 mm × 75 mm; 2.7 µm; Agilent InfinityLab, Las Rozas, Madrid, Spain) was used at 35 °C for compound separation following the gradient described previously [26]. The quantification was carried out with ten level calibration curves obtained from pure standards (delphinidin-3-*O*-glucoside: y = 1047.4x + 8.3518, R^2^: 0.99995; cyanidin-3-*O*-glucoside: y = 1217.5x + 3.2479, R^2^: 0.99996; petunidin-3-*O*-glucoside: y = 832.74x + 2.6594, R^2^: 0.99994; pelargonidin-3-*O*-glucoside: y = 1371.9x + 7.8528, R^2^: 0.99997; and malvidin-3-*O*-glucoside: y = 1011x + 6.8629, R^2^: 0.99996) (Sigma-Aldrich, Schnelldorf, Germany). The results were expressed as milligrams per gram of sample (mg/g, dw)

### 2.4. Determination of Total Polyphenols and Phenolic Families by QUENCHER Methodology

#### 2.4.1. Determination of Total Polyphenols by QUENCHER Methodology

The total polyphenol content (TPC) was determined in BVFF and BVE through the Fast Blue BB method [27,28] using the QUENCHER methodology [29]. Briefly, 0.4 mL of 0.1% Fast Blue BB (Sigma-Aldrich, Quentin Fallavier, France), 0.4 mL of 5% NaOH (Panreac, Barcelona, España), and 4 mL of distilled water were added to 1 ± 0.5 mg of the sample, followed by vortex homogenization after the addition of each reagent. After 45 min on an orbital shaker, the sample was centrifugated at 6500 rpm for 10 min and filtered; finally, the absorbance was measured at 420 nm in a UV-vis microplate reader (Synergy HTX, Biotek, Santa Clara, CA, USA). A gallic acid (Sigma-Aldrich, Quentin Fallavier, France) calibration curve was obtained with different concentrations from 0.625 to 160 µg/mL; hence, the results were expressed as milligrams of gallic acid equivalent per gram of sample (mg GAE/g dw).

#### 2.4.2. Determination of Hydroxybenzoic Acids by QUENCHER Methodology

The determination of hydroxybenzoic acid content (HBC) was carried out for BVFF and BVE, according to Bonoli et al. [30] using the QUENCHER methodology. Briefly, 0.5 mL of distilled water and 4 mL of 3% formic acid (Scharlau, Sentmenat, Spain) were added to a 1 ± 0.5 mg of the sample (freeze-dried fruit sample and optimized extract), homogenized by vortexing, and placed on an orbital shaker. After 15 min, the sample was centrifugated at 6500 rpm for 5 min and filtered, then the absorbance was measured at 280 nm in quartz cuvettes using a UV-vis microplate reader (Synergy HTX, Biotek, Santa Clara, CA, USA). A gallic acid (Sigma-Aldrich, Quentin Fallavier, France) calibration curve was obtained with different concentrations from 3.125 to 400 µg/mL, whereby the results were expressed as milligrams of gallic acid equivalent per gram of sample (mg GAE/g dw).

#### 2.4.3. Determination of Hydroxycinnamic Acids by QUENCER Methodology

The determination of hydroxycinnamic acid content (HCC) was made in BVFF and BVE, according to Bonoli et al. [30] using the QUENCHER methodology. In short, 0.5 mL of distilled water and 4 mL of methanol were added to 1 ± 0.5 mg of the sample (freeze-dried fruit sample and optimized extract), homogenized by vortexing, placed on an orbital shaker, and centrifugated after 15 min at 6500 rpm for 5 min; finally, the absorbance was measured at 320 nm in a UV-vis microplate reader (Synergy HTX, Biotek, Santa Clara, CA, USA). A ferulic acid calibration curve was obtained with different concentrations from 3.125 to 200 µg/mL; hence, the results were expressed as milligrams of ferulic acid equivalent per gram of sample (mg FAE/g dw).

#### 2.4.4. Determination of Flavonols by QUENCHER Methodology

The determination of flavonol content (FC) was carried out in BVFF and BVE, accordingly to Bonoli et al. [30] using the QUENCHER methodology. Briefly, 0.5 mL of distilled water and 4 mL of methanol were added to 1 ± 0.5 mg of the sample (freeze-dried fruit sample and optimized extract). The mixture was homogenized by vortex and placed on an orbital shaker for 15 min. After centrifugation for 15 min at 6500 rpm, the sample was filtered, and the absorbance was measured at 370 nm with a UV-vis microplate reader (Synergy HTX, Biotek, Santa Clara, CA, USA). Different concentrations from 3.125 to 250 µg/mL of quercetin (Scharlau, Sentmenat, Spain) were used to obtain a calibration curve; hence, the results were expressed in milligrams of quercetin equivalent per gram of sample (mg QE/g, dw).

### 2.5. Bioactive Properties of Berberis vulgaris and Its Optimized Extract

#### 2.5.1. Antioxidant Activity

Antioxidant activity was evaluated in BVFF and BVE using two different in vitro chemical methods, Folin–Ciocalteu and DPPH assays, both carried out through the QUENCHER methodology. In addition, an in vitro biological method was employed: the cell-based oxidative hemolysis inhibition assay (OxHLIA).

##### Folin–Ciocalteu Assay

The reduction of the Folin–Ciocalteu reagent (Scharlau, Sentmenat, Spain) generated by the samples was measured according to Del Pino-García et al. [23] with some modifications. Briefly, 0.2 mL of distilled water and 0.2 mL of Folin–Ciocalteu reagent were added to 1 ± 0.5 mg of the sample, homogenized by vortex, and placed on an orbital shaker; after 5 min, 4 mL of Na_2_CO_3_ (Sigma-Aldrich, Quentin Fallavier, France) and 5.6 mL of distilled water were added, the sample was vortexed again, and then it was replaced on the orbital shaker for 50 min. The mixture was then centrifugated for 5 min at 6500 rpm and filtered. Finally, the absorbance was measured at 750 nm in a UV-vis microplate reader (Synergy HTX, Biotek, Santa Clara, CA, USA). A gallic acid calibration curve was obtained with concentrations from 50 to 400 µg/mL; thus, results were expressed as milligrams of gallic acid equivalent per gram of sample (mg GAE/g sample, dw).

##### DPPH Assay

The scavenging capacity of 2,2-diphenyl-1-picriylhydrazyl (DPPH; Sigma-Aldrich, Schnelldorf, Germany) radical by the sample was determined according to Del Pino-García et al. [23] with some modifications. Concisely, 10 mL of DPPH reagent was added to 1 ± 0.5 mg of the sample, homogenized by vortex, and placed on an orbital shaker for 1 h. The sample was then centrifugated at 7000 rpm for 5 min and filtered. The absorbance was measured at 517 nm in a UV-vis microplate reader (Synergy HTX, Biotek, Santa Clara, CA, USA). A Trolox calibration curve was obtained from 6.25 to 400 µg/mL; hence, the results were expressed as milligrams of Trolox equivalent per gram of sample (mg TE/g, dw).

##### Antihemolytic Activity: OxHLIA Assay

The antihemolytic activity assay was performed according to Lockowandt et al. [31]. Extract solutions (fruit: 0.084–2.0 mg/mL; optimized extract: 0.03–2.0 mg/mL) in PBS (Sigma-Aldrich, Madrid, Spain), distilled water (for complete hemolysis), and Trolox (7.81–250 µg/mL in PBS), as the positive control, were mixed with 400 µL of erythrocyte solution (2.8%, *v*/*v*; 200 µL in PBS, pH 7.4) and pre-incubated at 37 ± 0.5 °C with shaking. After 10 min, 200 µL of 2,2′-azobis(2-methylpropionamidine) dihydrochloride (AAPH, 160 mM in PBS) was added. Optical density was measured at 690 nm every 10 min using an Elx800 microplate reader (Bio-Tek Instruments, Winooski, VT, USA) until complete hemolysis. Results were expressed as IC_50_ values (µg/mL) for a Δt of 60 min, obtained by correlating the extract concentrations with Δt values (min) (which resulted from the half-hemolysis time (Ht50) obtained graphically using GraphPad Prism 8, San Diego, CA, USA) from the hemolytic curves of each extract.

#### 2.5.2. Antibacterial Activity

The samples were tested against eight different bacterial strains relevant to food safety, five Gram-negative—*Enterobacter cloacae* (ATCC 49741), *Escherichia coli* (ATCC 25922), *Pseudomonas aeruginosa* (ATCC 9027), *Salmonella enterica serotype Enteritidis* (ATCC 13076), and *Yersinia enterocolitica* (ATCC 8610)—and three Gram-positive—*Bacillus cereus* (ATCC 11778), *Listeria monocytogenes* (ATCC 19111), and *Staphylococcus aureus* (ATCC 25923). All microorganisms were purchased from Frilabo, Porto, Portugal. The minimum inhibitory concentration (MIC) was determined according to Pires et al. [32]. Before analysis, bacterial cultures were incubated at 37 °C in fresh medium for 24 h to generate exponential growth. Samples were dissolved in 5% dimethyl sulfoxide (DMSO) and sterile water to a final concentration of 20 mg/mL, in duplicate. Then, 90 µL of this stock solution was added to 100 µL of Tryptic Soy Broth (TSB) in the first well, with serial dilutions (10 to 0.03125 mg/mL) prepared in the remaining wells; a bacteria inoculum (standardized at 1.5 × 10^5^ colony-forming units (CFUs)/mL) was added to each well. Negative controls were prepared with TSB and the sample, and positive controls were prepared with TSB and each inoculum, as well as with medium, antibiotics, and bacteria. Methicillin was used for *Staphylococcus aureus*; for the other bacteria, ampicillin and streptomycin were used. The microplates were incubated at 37 °C for 24 h; finally, 40 µL of 0.2 mg/mL *p*-iodonitrotetrazolium chloride (INT) was added and incubated at 37 °C for 30 min to determine the MIC, which was defined as the lowest concentration that inhibited visible bacteria growth, shown by a change from yellow to pink in the viable microorganisms. Finally, minimum bactericidal concentration (MBC) was determined by subculturing wells without color change onto blood agar (7% sheep blood) and incubating at 37 °C for 24 h. MBC was defined as the lowest concentration (mg/mL) required to achieve bacterial death.

#### 2.5.3. Antifungal Activity

Antifungal activity was determined according to Heleno et al. [33]. The samples were tested against two fungal strains: *Aspergillus fumigatus* (ATCC 204305) and *Aspergillus brasiliensis* (ATCC 16404), obtained from Frilabo, Porto, Portugal. Fungal cultures were incubated at 25 °C for 72 h. For the determination of the antifungal activity, 100 µL of a spore suspension with an approximate concentration of 1.0 × 10^5^ obtained by washing the fungal spores from the surface of agar plates (with 0.85 sterile saline containing 0.1% Tween 80 (*v*/*v*)) were placed in the wells. The sample was dissolved in 5% DMSO and sterile water to a concentration of 20 mg/mL, and 90 µL of the solution was added in duplicate to the first well with 100 µL of Malt Extract Broth (MEB). Serial dilutions (10 to 0.03125 mg/mL) were prepared in the remaining wells. The lowest concentration without visible growth after 72 h at 26 °C was defined as the minimum inhibitory concentration (MIC). The minimum fungicidal concentration (MFC) was determined after subculturing on MEB and incubating for a further 72 h at 26 °C, and was defined as the lowest concentration with no visible growth, indicating 99.5% the original fungi have been killed. The commercial fungicide ketoconazole was used as a positive control.

### 2.6. Statistical Analysis

All the analyses were carried out in triplicate, and the results are presented as means values ± standard deviation. Student’s *t* test was used for comparison and identification of the significant differences among the sets of means. All the analyses were performed using the XLSTAT program (Addinsoft, 2024. New York, NY, USA).

## 3. Results and Discussion

### 3.1. Physicochemical Characterization of Berberis vulgaris Fresh Fruit Sample (BVF)

As a first approach to *B. vulgaris* wild fruit evaluation, the physicochemical properties of BVF were analyzed in terms of moisture, pH, titratable acidity, and Brix degrees. High moisture was evidenced with a content of 68.02 g of water/100 g of fruit, which is consistent with the range of 58.39 to 71.53% reported for eight different varieties of the same species [34]. These fruits also showed an optimal ripening index, with a Brix value of 12.89 and an acidic pH around 2.94, which are in accordance with those previously reported for fruits collected from different regions of Turkey [9,34]. Moreover, 415.36 mL of NaOH was needed to neutralize the acids present in 100 g of fruit.

Regarding the color, the L* (19.84 ± 0.12) and b* (5.69 ± 0.09) parameters were similar to those previously reported by other authors for twenty-five different genotypes; however, the a* (19.89 ± 1.93) parameter was remarkably higher, evidencing in this way a dark red wine color, confirmed by a chroma (C*) of 20.69 and hue (h) of 0.28 [35,36].

### 3.2. Determination of the Optimal Extraction Conditions Through RSM

According to the literature, several factors can interfere with the extraction of anthocyanin from different natural matrices in an independent way. Consequently, preliminary research was conducted in order to determine their experimental domain for the proposed food matrix, the *Berberis vulgaris* fruit fraction (BVFF; *B. vulgaris* fruits without seeds); this means that four independent factors were studied across various level ranges. Both pH and S/L ratio factor were evaluated at two levels, 3 and 6 for pH and 16.66 and 33.33 for S/L ratio, ultrasound power at three levels, 250, 400 and 500 W, and extraction time at five levels, 2.5, 5, 10, 15, and 20 min. As a result of the different variable combinations, a total of 60 assays were obtained, which were carried out in duplicate (n = 120), and for each of them, the total monomeric anthocyanin content as well as the color characteristics were determined (Table 3).

The response variables exhibited significant variability; in the case of the total anthocyanin content, values ranging from 885.32 to 1254.58 mg cya-3-glu/g were obtained. The color parameters showed a more extensive variation, with values from 19.92 to 43.8 for the L* parameter, 19.82 to 48.47 for the a* parameter, and from 8.57 to −9.18 for the b* parameter. This color variability is further reflected in chroma (48.57 to 20.51) and hue values (10.58 to −18.02). Due to the pronounced variation in color and the influence of other factors such as pH, the extraction conditions were optimized to prioritize maximum anthocyanin yield, focusing on total anthocyanin content data.

The experimental results (Table 3) were utilized to construct a response surface model (RSM), which allowed us to extrapolate the findings beyond the specific tested conditions and to delve deeper into the factors influencing anthocyanin concentration. This process involved calculating the coefficients of the second-order polynomial equation (Equation (3)), resulting in the following model (Equation (4)):

(4) $$Y=865.616+67.0892X_{1}+3.62955X_{2}-16.9832X_{3}+9.91258X_{4}-\;2.89838X_{1}^{2}-0.030752X_{2}^{2}+0.106308X_{3}^{2}-0.208484X_{4}^{2}-0.297198X_{1}X_{2}-\;0.424065X_{1}X_{3}-1.76301X_{1}X_{4}+0.0929015X_{2}X_{3}+0.0584702X_{2}X_{4}+0.218318X_{3}X_{4}$$

where X_1_ refers to pH, X_2_ to the ultrasound power used, X_3_ to extraction time, and X_4_ to the solid/liquid ratio, while Y represents the anthocyanin content. The values of the coefficient of determination (R^2^) and adjusted R^2^ were 0.997115 and 0.996153, respectively, demonstrating an excellent fit of the model.

**Figure 1:**
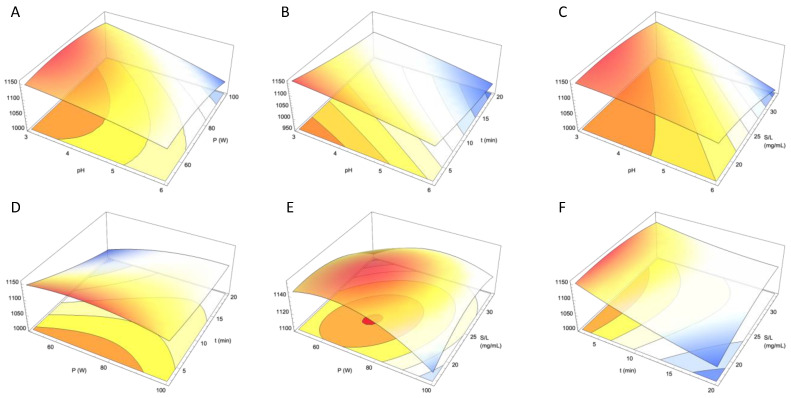
Response surface graphs of the combined effect of the independent variable (pH, ultrasound power used (P), extraction time (t), and solid/liquid ratio (S/L)) on total anthocyanin content (in mg cya-3-glu/g; Y-axis). Excluded variables in each graph were positioned at their optimal values. (**A**) combined effect of pH (X-axis) and P (Z-axis); (**B**) combined effect of pH (X-axis) and t (Z-axis); (**C**) combined effect of pH (X-axis) and S/L (Z-axis); (**D**) combined effect of P (X-axis) and t (Z-axis); (**E**) combined effect of P (X-axis) and S/L (Z-axis); (**F**) combined effect of t (X-axis) and S/L (Z-axis).

The magnitude of the RSM coefficients indicates the strength and type of relationship each variable has with the response variable (Y). Larger absolute coefficients indicate a more significant impact on the results, considering the magnitudes of the variables. In this model, pH (X_1_) and its linear term exert the most substantial influence on the response, followed by the linear terms of time (X_3_) and the solid/liquid ratio (X_4_). Additionally, the interaction between pH and S/L ratio (X_1_ and X_4_) can also influence anthocyanin extraction and, therefore, the anthocyanin content in the extract. Statistical analysis of the individual parameters, presented in Supplementary Table S1, highlights the significant influence of pH (X_1_) and the combined effect of pH and solid/liquid ratio (X_1_ and X_4_) while showing the lower significance of other parameters. Notably, the linear term for time shows a high negative value, suggesting that anthocyanin content decreases as extraction time increases (Figure 1).

Interestingly, the linear effect of pH is counteracted by quadratic and interaction terms, resulting in an overall decrease in anthocyanin content as pH increases. However, the effects of the other two variables on the response are less straightforward, showing a more complex behavior, indicating intricate dynamics among the terms.

Using the RSM model, the optimal experimental conditions for maximizing anthocyanin content within our experimental range were identified. According to the optimization procedure, the maximum anthocyanin content of 11.56 mg cyanidin-3-glucoside/g was achieved at pH 3, with an ultrasound power of 345 W, an extraction time of 2.5 min, and a solid/liquid ratio of 22.12 mg/mL.

### 3.3. Anthocyanin Characterization in Berberis vulgaris Freeze-Dried Fruit Fraction (BVFF) and Optimized Colorant Extract (BVE)

Regarding anthocyanin content, BVFF presented a total monomeric anthocyanin content (expressed as cyanidin-3-*O*-glucoside equivalent, measured by the QUENCHER methodology) of 11.49 ± 0.27 mg cyanidin-3-glucoside/g (dw), while BVE presented a total amount of 12.42 ± 0.54 mg cya-3-glu/g (dw). This value was in accordance with those previously reported by other authors, such as the range between 12.07 and 36.05 reported for an extract from seedless *B. vulgaris* wild fruits from Iran [37]. Additionally, other studies have reported TAC values of 602 and 785 mg cya-3-glu/L in aqueous and alcoholic extracts, respectively [38].

Moreover, BVE demonstrated comparable or superior anthocyanin content to other optimized extracts. For instance, an optimized extract of purple corn (*Zea Mays* L.), obtained through infrared irradiation, presented a value of 14 mg cyanidin-3-glucoside/g, dw [39]. Similarly, an extract of jaboticaba fruits recorded a TAC of 14.58 mg/g (dw) [40]. An extract of sweet cherry (*Prunus avium*) displayed a TAC of 12.2 mg cyanidin-3-glucoside/g (dw) [41]. In contrast, an extract of blackberry (*Rubus fruticosus*) exhibited a lower TAC of 4.31 mg cyanidin-3-glucoside/g (dw) [42].

**Figure 2:**
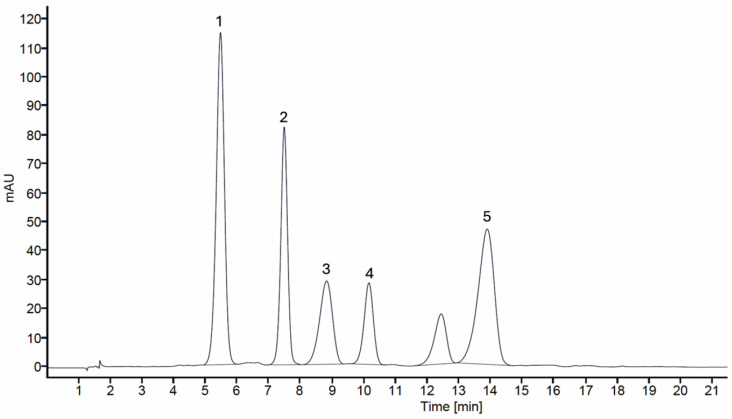
Chromatographic profile at 520 nm of the anthocyanin compounds in *Berberis vulgaris* optimized colorant extract (1: delphinidin-3-*O*-glucoside; 2: cyanidin-3-*O*-glucoside; 3: petunidin-3-*O*-glucoside; 4: pelargonidin-3-*O*-glucoside; 5: malvidin-3-*O*-glucoside).

The individual anthocyanin profile of BVE was also characterized, identifying five anthocyanins, as can be seen in Figure 2 (chromatographic profile of anthocyanins found in wild *B. vulgaris* optimized extract). In addition, Table 4 shows detailed information on the peak characteristics and provides a tentative identification of each individual anthocyanin, as well as their quantification, in both BVFF and BVE.

Peaks 1 (delphinidin-3-*O*-glucoside), 2 (cyanidin-3-*O*-glucoside), 3 (petunidin-3-*O*-glucoside), 4 (pelargonidin-3-*O*-glucoside), and 5 (malvidin-3-*O*-glucoside) were identified through a comparison with authentic standards and confirmed by the fragmentations observed using mass spectrometry. Peak 1 ([M]^+^ at *m*/*z* 465) presented a unique MS^2^ fragment at 303, characteristic of delphinidin aglycone. Peak 2 presented [M^+^] at *m*/*z* 449 and a unique MS^2^ fragment at *m*/*z* 287, characteristic of cyanidin aglycone. Peak 3 ([M]^+^ at *m*/*z* 524) presented an MS^2^ fragment at *m*/*z* 317, characteristic of petunidin aglycone. Peak 4 ([M]^+^ at *m*/*z* 433) presented an MS^2^ fragment at *m*/*z* 271, characteristic of pelargonidin aglycone. Lastly, peak 5 presented a protonated ion at *m*/*z* 493 and a unique MS^2^ fragment at *m*/*z* 331, characteristic of malvidin aglycone. All these molecules showed neutral losses of 162 Da, evidencing hexose moieties linked to the aglycone. While the presence of delphinidin-3-*O*-glucoside, cyanidin-3-*O*-glucoside, and pelargonidin-3-*O*-glucoside has been previously reported in the variety seedless barberry [43], this study reports for the first time the presence of petunidin-3-*O*-glucoside and malvidin-3-*O*-glucoside, along with the individual quantification of all five identified anthocyanins.

Regarding the quantification, it is clear that the content of the five individual anthocyanins was significantly higher in the extract than in the fruit, with delphinidin-3-*O*-glucoside being the most abundant in both cases, reaching 3.54 mg/g (dw) in BVFF and 5.27 g/g (dw) in BVE, closely followed by malvidin-3-*O*-glucoside and cyanidin-3-*O*-glucoside. Moreover, pelargonidin-3-*O*-glucoside was the least abundant anthocyanin, with 0.67 mg/g (dw) in BVFF and 1.00 mg/g (dw) in BVE. Moreover, delphinidin-3-*O*-glucoside presented the highest increment (increase of 0.95 mg/g), followed closely by malvidin-3-*O*-glucoside, which increased by 0.81 mg/g. Overall, a 1.1-fold increase in total anthocyanin content was noted in BVE compared to BVFF.

The richness of this colorant extract was not only evident due to its high anthocyanin concentration but also because of the diversity of anthocyanins present, where with just five anthocyanins in its composition, it encompasses derivatives of delphinidin, cyanidin, petunidin, pelargonidin, and malvidin. To date, the individual anthocyanin quantification of *B. vulgaris* has not been reported, limiting direct comparisons with previous data. Nonetheless, similar berries rich in anthocyanins have exhibited similar or even lower contents of individual anthocyanins. For instance, a blackberry extract displayed a similar composition to *B. vulgaris* with cyanidin-3-*O*-glucoside (1.63 mg/g extract, dw), cyanidin-3-*O*-rutinoside (0.51 mg/g extract, dw), and cyanidin-3-*O*-arabinoside (1.45 mg/g extract, dw) as the predominant anthocyanins, reaching a total of 26.17 mg/g of extract [44,45]. Similarly, in *Vaccinium myrtillus* L., 4.47 mg of delphinidin-3-*O*-glucoside, 4.02 mg of cyanidin-3-*O*-glucoside, 1.99 mg of petunidin-3-*O*-glucoside, and 1.02 mg of malvidin-3-*O*-glucoside/g of extract were quantified [29]. Likewise, an optimized extract of *Berberis crataegina*, obtained through UAE, revealed a similar composition, with cyanidin-3-O-glucoside and cyanidin-3-*O*-rutinoside being the predominant anthocyanins, reaching a total of 291.2 mg/g of identified anthocyanins [46]. Furthermore, an extract of grape by-product presented an individual anthocyanin profile comprising delphinidin-3-*O*-glucoside (0.47 mg/g), cyanidin-3-*O*-glucoside (0.27 mg/g), petunidin-3-*O*-glucoside 0.66 mg/g), peonidin-3-*O*-glucoside (2.90 mg/g), and malvidin-3-*O*-glucoside (1.68 mg/g) [47].

### 3.4. Total Polyphenols and Phenolic Families in Berberis vulgaris Freeze-Dried Fruit Fraction (BVFF) and Optimized Colorant Extract (BVE)

The content of phenolic compounds found in BVFF and BVE is shown in Table 5. The hydroethanolic extraction process carried out was highly efficient, extracting the majority of the phenolic compounds, resulting in an extract rich not only in anthocyanins but also in various other phenolic compounds.

The total phenolic content (TPC) was notably elevated, with a value of 290.72 mg GAE/g, nearly double the content determined in BVFF (159.90 mg GAE/g). In addition, this TPC value surpassed previously reported findings by other researchers. Specifically, it exceeded values of 100.86 mg GAE/g of optimized colorant extract (dw), obtained by ultrasonic water bath extraction for 30 min at 50 °C [48], as well as the value of 10.84 mg GAE/g of extract (dw) obtained through a similar extraction method at 60 °C [49]. Moreover, these results are in accordance with the TPC range of 100 to 280 mg/g (dw) of extract observed in fruits subjected to decoction for 30 min collected in Malaysia [50] and fall within the range of 184.1 to 291.22 mg GAE/g of extract (dw) obtained through decoction with water and ethanol/water in fruits collected in Iran [38].

Additionally, when compared to extracts from other fruits with high content of bioactive compounds, the superiority of BE becomes evident. For example, an optimized extract of purple corn (*Zea mays* L.) presented a TPC of 37 mg GAE/g, dw [39]. Similarly, an optimized extract of blackthorn (*Prunus spinosa* L.) presented a value of 32.2 mg GAE/g, dw [51]. Furthermore, an optimized extract of jaboticaba achieved a TPC of 199.34 mg GAE/g (dw) [40].

Hydroxybenzoic acids (HBC) emerged as the predominant phenolic family, with values of 81.74 mg GAE/g (dw) in BVE and 47.30 mg GAE/g (dw) in BVFF, followed by hydroxycinnamic acids (HCC, at 46.52 mg FAE/g (dw) in BVE). In contrast, FC represented the least abundant phenolic family with a concentration of 10.00 mg FAE/g of extract (dw) in BVE.

To date, there have been no previous reports determining the presence of these phenolic families in this fruit; however, the richness and superiority in content of phenolic compounds of *B. vulgaris* samples becomes evident when compared to other berries. For instance, extracts obtained from *Vaccinium myrtillus* L. (bilberry) reported contents of hydroxybenzoic acids between 0.033 and 0.072 mg GAE/g (dw), hydroxycinnamic acids ranging from 1.13 to 2.31 mg chlorogenic acid equivalent (CAE)/g (dw), and flavonols between 0.54 and 1.30 mg rutin equivalent (RE)/g. Similarly, *Ribes nigrum* L. (cultivars Ojebyn and Titan; blackcurrant) presented values of hydroxybenzoic acids between 0.06 and 0.12 mg GAE/g (dw), hydroxycinnamic acids between 0.58 and 0.93 mg CAE/g (dw), and flavonols between 0.72 and 0.87 mg RE/g (dw). In *Vaccinium vitis-idaea* L. (cowberry), concentrations of hydroxybenzoic acids between 0.10 and 0.16 mg GAE/g (dw), hydroxycinnamic acids between 0.46 and 0.70 mg CAE/g (dw), and flavonols between 1.02 and 1.53 mg RE/g (dw) were reported [29,52].

### 3.5. Bioactive Properties of B. vulgaris Freeze-Dried Fraction Fruit Sample (BVFF) and Its Optimized Colorant Extract (BVE)

#### 3.5.1. Antioxidant Activity

For the characterization of the bioactive properties of *B. vulgaris* samples, BVFF and BVE, the antioxidant, antimicrobial, and antifungal activity were evaluated (Table 5). Antioxidant activity was determined through two in vitro chemical methods, the Folin–Ciocalteu and DPPH, and one in vitro biological method, the OxHLIA assay.

Although the Folin–Ciocalteu method is commonly used for the measurement of total phenolic compounds, the Folin–Ciocalteu reagent can be reduced by phenolic compounds as well as by other reducing agents present in vegetal samples such as sugars, vitamins, organic acids, etc. This makes it indicative of antioxidant capacity.

Both the BVFF and BVE presented higher antioxidant activity through single-electron transfer, evaluated by the DPPH method, with values of 111.37 mg TE/g dw for BVE and 76.30 mg TE/g dw for BVFF. These values were higher compared to those obtained through hydrogen atom transfer, evaluated by the Folin–Ciocalteu method, which yield values of 88.03 mg GAE/g for BVE and 60.99 mg GAE/g for BVFF. In addition, it is important to highlight that BVE presented higher antioxidant activity than BVFF through both methodologies, evidencing an increase above 1.4-fold, as expected, due to its higher concentration of phenolic compounds.

In a previous study, an antioxidant capacity of 50.85 mg TE/g of extract using DPPH was reported for an extract of *B. vulgaris* fruits from Italy, obtained by ultrasound water bath extraction at 50 °C for 30 min [48]. This value was lower than those found in the present study. Another study using an ethanolic extract obtained by ultrasonic water bath extraction at 60 °C for 30 min from *B. vulgaris* fruits collected in Turkey reported an IC_50_ of 0.14 mg/mL to achieve 50% scavenging of the DPPH radical [49]. Similarly, *B. vulgaris* fruits from different locations in Turkey displayed DPPH values ranging from 11.92 to 40.44% in extracts obtained by maceration [34]. Furthermore, aqueous and ethanolic extracts from Iranian fruits showed DPPH values between 19.88 and 35.17% [43].

Compared to other extracts from matrices with high antioxidant capacity, the obtained extract, BVE, demonstrated superiority. For instance, an extract of blackberry (*Rubus fruticosus*) presented a value of 83.00 mg TE/g (dw) through the DPPH methodology [42]

Furthermore, the BVFF sample showed better antioxidant capacity using the OxHLIA assay, with an IC_50_ of 60 µg/mL compared to BVE, which presented an IC_50_ of 125 µg/mL, evidencing that the extract required a higher concentration to protect the erythrocyte population from hemolysis. The results obtained are in agreement with those previously reported by other authors such as Moldovan et al. [48], who found an IC_50_ of 76 µg/mL for an ethanolic extract of *B. vulgaris* fruits from Italy. Moreover, the values obtained for the optimized extracts are consistent with those previously reported for other similar samples, such as red raspberry extract (*Rubus idaeus* L., Fam. Rosaceae), which presented IC_50_ values of 449 and 520 µg/mL for extracts obtained by heat-assisted and ultrasound-assisted extraction, respectively [53].

#### 3.5.2. Antibacterial Activity

Both wild *B. vulgaris* freeze-dried fruit fraction sample (BVFF) and its colorant extract (BVE) were tested against five Gram-negative bacteria and three Gram-positive bacteria, all of which are responsible for foodborne diseases. BVE exhibited a notable improvement in efficacy compared to the fruit, evidenced by a reduction in the minimum inhibitory concentration (MIC) for all tested bacteria and the presence of bactericidal activity, which was not observed in BVFF. Specifically, BVE stood out for its inhibitory capacity against all the bacteria tested, as well as its bactericidal capacity against all of them, with the exception of *Listeria monocytogenes*. BVE was especially effective against *Salmonella enterica* and *Bacillus cereus*, with an MIC of 1.25 mg/mL in both cases, as well as *Escherichia coli* and *Staphylococcus aureus*, with an MIC of 2.5 mg/mL. Moreover, BVE presented a minimum bactericidal concentration (MBC) of 10 mg/mL for *Enterobacter Cloacae*, *Escherichia coli*, *Pseudomonas aeruginosa*, and *Bacillus cereus.* The BVFF sample did not show bactericidal activity against any of the bacteria tested; however, it presented inhibitory activity against all the bacteria tested, standing out with MIC values of 2.5 and 5 mg/mL for *Salmonella enterica* and *Escherichia coli*, respectively.

Both BVFF and BVE showed improved antibacterial activity compared to previous findings. For instance, Gidik et al. [49] reported MIC values of 16 mg/mL against *E. coli* and *Y. enterocolitica* and 32 mg/mL against *S. aureus*, *B. cereus*, and *P. aeruginosa*. Moreover, the antibacterial activity of *B. vulgaris* extract has been reported against other bacteria associated with food contamination, such as *Enterococcus faecium*, *Streptococcus salivarus*, *Pseudomonas fluorescens*, *Vibrio parahaemolyticus*, *Aeromonas cavia*, and *Salmonella Typhimurium*, as well as bacteria associated with dental caries, such as *Streptococcus sobrinus*, *Streptococcus sanguis*, and *Streptococcus mutans* [8,49,54].

Nonetheless, BVE showed significantly greater antibacterial activity compared to extracts from other vegetal samples rich in anthocyanins with demonstrated antibacterial activity. For example, passion fruit (*Passiflora edulis* Sims) extract presented MIC values ranging between 4 and 8 mg/mL against *S. aureus*, *L. monocytogenes*, *E. coli*, *E. cloacae*, and *S.* Typhimurium [55]. Similarly, an optimized extract of jaboticaba presented a 22% increase in microbial inhibition of *E. coli* at a concentration of 50 mg/mL [40].

#### 3.5.3. Antifungal Activity

In terms of antifungal activity, both BVFF and BVE presented strong inhibitory activity against the two fungi tested, particularly *Aspergillus brasiliensis*, where BVFF presented an MIC of 2.5 mg/mL and BVE had an MIC of 5 mg/mL. Moreover, both samples demonstrated an MIC of 10 mg/mL against *Aspergillus fumigatus.* To the best of the authors’ knowledge, no previous studies have been published regarding the antifungal capacity of *B. vulgaris* fruit or its extract. However, the results in this study indicate a comparable or slightly enhanced antifungal activity compared to other fruits with high anthocyanin content. For instance, *Vaccinium myrtillus* L. presented an MIC of 10 mg/mL against *Aspergillus fumigatus* and <10 mg/mL against *Aspergillus brasiliensis* [27].

## 4. Conclusions

In this work, *Berberis vulgaris* wild fresh fruits (BVFs) were characterized in terms of physicochemical properties, as well as bioactive compounds, evidencing an intense red color, and relevant anthocyanin content. Optimal extraction conditions for *B. vulgaris* freeze-dried fruit fraction (BVFF) through ultrasound-assisted extraction methodology were determined through response surface methodology (RSM). The BVFF was subjected to different extraction conditions in terms of extraction time (t), pH of the extraction solvent, ultrasound power (P), and solid/liquid ratio (S/L). The response criteria used were total anthocyanin content and color parameters, specifically chroma (C*) and hue (h). Different optimal conditions were found for each response criteria, evidencing that the extraction conditions significantly influenced the responses studied. Furthermore, the proposed extraction models presented high values of adjusted determination coefficient (r^2^ > 0.97).

A colorant extract rich in anthocyanins was obtained through the optimal conditions determined for total anthocyanin content (t: 2.5 min; pH: 3; P: 345 W and S/L: 22.12), yielding 290.72 mg of total phenolic content/g of extract (dw). The optimized colorant extract (BVE) was predominantly composed of hydroxybenzoic acids (81.74 mg/g extract). A total monomeric anthocyanin content of 12.42 mg/g of extract was determined, with delphinidin-3-*O*-glucoside being the predominant individual anthocyanin characterized. These values were significantly higher than those obtained for BVFF, evidencing the obtention of an extract rich in bioactive compounds and an optimal extraction of the anthocyanins present in *B. vulgaris* fruits.

Regarding the evaluated bioactivities, BVE presented stronger antioxidant activity compared to BVFF, especially through the DPPH assay. It also showed improved antibacterial activity, especially against *Salmonella enterica*, *Escherichia coli*, and *Bacillus cereus*. Furthermore, BVE also presented strong antifungal properties.

Moreover, BVE demonstrated a remarkable richness in anthocyanins, presenting a high diversity with five distinct anthocyanidins in its composition (delphinidin, cyanidin, petunidin, pelargonidin, and malvidin). These compounds contribute to its characteristic intense red color. In addition, BVE showed higher anthocyanin content compared to other fruits renowned for their high levels of these pigment molecules, such as blackberry and bilberry. It also exhibited superior levels of other bioactive compounds, resulting in enhanced antioxidant activity and antimicrobial activity against foodborne bacteria and fungi.

In conclusion, this work reports an optimized colorant extraction system for obtaining an extract rich in anthocyanins from *Berberis vulgaris*, which also presents remarkable content of other bioactive compounds and exhibits potent bioactive capacities. This extract holds significant potential as a natural food colorant to be used in many different types of food formulations. The extraction conditions and the chemical composition have been filed to the Spanish Patent Office under patent ES-2990137 A1.

## Figures and Tables

**Table 1:** *Berberis vulgaris* L. fruits (BVFs) and their collection sites and coordinates.

	Location 1	Location 2
	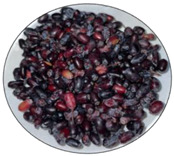	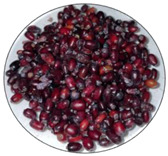
Location	Dehesa de Carrascosa, Cuenca (Spain)	Laguna de El Tobar Beteta, Cuenca (Spain)
Latitude	40°35′14.8″ N	40°32′21.1″ N
Longitude	2°08′49.9″ W	2°03′19.5″ W

**Table 2:** Experimental design for the optimization of the anthocyanin extraction through ultrasound-assisted extraction.

Code	pH	P (W)	t (min)	S/L (mg/mL)	Code	pH	P (W)	t (min)	S/L (mg/mL)
1	3	250	2.5	33.33	31	6	250	2.5	33.33
2	3	250	5	33.33	32	6	250	5	33.33
3	3	250	10	33.33	33	6	250	10	33.33
4	3	250	15	33.33	34	6	250	15	33.33
5	3	250	20	33.33	35	6	250	20	33.33
6	3	400	2.5	33.33	36	6	400	2.5	33.33
7	3	400	5	33.33	37	6	400	5	33.33
8	3	400	10	33.33	38	6	400	10	33.33
9	3	400	15	33.33	39	6	400	15	33.33
10	3	400	20	33.33	40	6	400	20	33.33
11	3	500	2.5	33.33	41	6	500	2.5	33.33
12	3	500	5	33.33	42	6	500	5	33.33
13	3	500	10	33.33	43	6	500	10	33.33
14	3	500	15	33.33	44	6	500	15	33.33
15	3	500	20	33.33	45	6	500	20	33.33
16	3	250	2.5	16.66	46	6	250	2.5	16.66
17	3	250	5	16.66	47	6	250	5	16.66
18	3	250	10	16.66	48	6	250	10	16.66
19	3	250	15	16.66	49	6	250	15	16.66
20	3	250	20	16.66	50	6	250	20	16.66
21	3	400	2.5	16.66	51	6	400	2.5	16.66
22	3	400	5	16.66	52	6	400	5	16.66
23	3	400	10	16.66	53	6	400	10	16.66
24	3	400	15	16.66	54	6	400	15	16.66
25	3	400	20	16.66	55	6	400	20	16.66
26	3	500	2.5	16.66	56	6	500	2.5	16.66
27	3	500	5	16.66	57	6	500	5	16.66
28	3	500	10	16.66	58	6	500	10	16.66
29	3	500	15	16.66	59	6	500	15	16.66
30	3	500	20	16.66	60	6	500	20	16.66

**Table 3:** Independent variable used in each test of the experimental design and the results obtained in each response criteria evaluated.

	Independent Variables Evaluated						
Code	TAC (mg cya-3-glu/g)	L*	a*	b*	C*	h	RGB
1	10.58 ± 0.04	22.79 ± 0.93	47.32 ± 0.96	3.72 ± 0.44	47.48 ± 0.68	4.50 ± 0.69	
2	10.07 ± 0.12	22.16 ± 1.13	46.96 ± 1.42	4.81 ± 0.63	47.21 ± 1.01	5.87 ± 1.08	
3	10.79 ± 0.02	22.17 ± 1.69	46.89 ± 0.89	4.76 ± 1.36	47.16 ± 0.52	5.81 ± 1.62	
4	9.87 ± 0.36	22.70 ± 0.01	46.77 ± 0.95	4.35 ± 0.46	46.98 ± 0.80	5.30 ± 0.90	
5	10.76 ± 0.38	22.93 ± 0.22	46.35 ± 1.05	3.91 ± 0.20	46.51 ± 0.82	4.82 ± 0.59	
6	11.03 ± 0.09	22.15 ± 1.16	47.21 ± 1.08	4.83 ± 0.43	47.46 ± 0.76	5.85 ± 0.66	
7	10.96 ± 0.01	21.83 ± 1.72	46.71 ± 1.56	5.20 ± 1.23	47.03 ± 1.04	6.39 ± 1.70	
8	11.25 ± 0.04	22.28 ± 1.29	47.57 ± 1.03	4.32 ± 0.61	47.79 ± 0.70	5.21 ± 0.78	
9	11.00 ± 0.01	22.00 ± 1.77	46.80 ± 0.82	4.83 ± 1.50	47.08 ± 0.44	5.91 ± 1.90	
10	10.89 ± 0.09	22.17 ± 1.25	46.41 ± 0.05	5.06 ± 0.63	46.70 ± 0.19	6.22 ± 0.87	
11	12.17 ± 0.75	21.63 ± 1.79	47.18 ± 1.48	5.41 ± 1.10	47.53 ± 0.95	6.58 ± 1.50	
12	11.96 ± 0.63	21.46 ± 1.86	46.93 ± 1.20	5.79 ± 1.37	47.33 ± 0.68	7.07 ± 1.81	
13	11.44 ± 0.17	22.22 ± 1.60	46.88 ± 0.42	4.58 ± 1.07	47.12 ± 0.21	5.59 ± 1.34	
14	11.35 ± 0.40	21.79 ± 2.01	47.04 ± 1.29	5.66 ± 2.03	47.43 ± 0.73	6.90 ± 0.63	
15	11.56 ± 0.81	21.23 ± 1.50	46.67 ± 1.03	7.15 ± 0.69	47.22 ± 0.63	8.72 ± 1.01	
16	12.15 ± 0.31	29.04 ± 1.76	46.10 ± 0.03	−5.75 ± 0.64	46.47 ± 0.31	−7.10 ± 0.76	
17	11.69 ± 0.06	29.01 ± 1.73	46.44 ± 0.51	−5.76 ± 0.84	46.81 ± 0.58	−7.06 ± 1.02	
18	11.58 ± 0.38	28.95 ± 2.49	46.31 ± 0.72	−5.45 ± 0.95	46.65 ± 0.51	−6.72 ± 1.33	
19	10.68 ± 0.07	29.73 ± 1.18	45.52 ± 0.20	−6.53 ± 0.41	45.99 ± 0.32	−8.16 ± 0.74	
20	10.09 ± 0.47	30.13 ± 1.64	45.06 ± 0.48	−6.70 ± 0.86	45.56 ± 0.48	−8.46 ± 1.04	
21	11.35 ± 0.31	28.28 ± 1.02	46.90 ± 0.20	−5.55 ± 0.75	47.23 ± 0.27	−6.74 ± 0.96	
22	10.60 ± 0.01	29.29 ± 1.55	45.88 ± 0.13	−6.31 ± 0.80	46.31 ± 0.34	−7.83 ± 0.93	
23	11.21 ± 0.60	29.55 ± 1.52	45.29 ± 0.23	−6.16 ± 0.50	45.71 ± 0.24	−7.75 ± 0.66	
24	10.28 ± 0.52	30.25 ± 1.23	44.42 ± 0.14	−6.23 ± 0.45	44.86 ± 0.29	−7.98 ± 0.56	
25	10.43 ± 0.90	29.35 ± 0.45	45.64 ± 1.33	−6.16 ± 0.36	46.06 ± 1.11	−7.68 ± 0.50	
26	10.65 ± 0.18	28.40 ± 1.15	46.89 ± 0.54	−5.42 ± 0.70	47.20 ± 0.53	−6.59 ± 0.62	
27	10.58 ± 0.05	28.68 ± 1.31	46.37 ± 0.53	−5.60 ± 0.44	46.71 ± 0.55	−6.88 ± 0.56	
28	10.87 ± 0.53	28.81 ± 1.56	46.40 ± 0.03	−5.80 ± 0.71	46.77 ± 0.34	−7.12 ± 0.97	
29	10.15 ± 0.36	29.31 ± 1.24	45.48 ± 0.11	−5.47 ± 0.45	45.82 ± 0.51	−6.87 ± 0.66	
30	10.73 ± 0.40	28.65 ± 1.41	46.21 ± 0.02	−4.97 ± 0.53	46.48 ± 0.37	−6.14 ± 0.54	
31	10.76 ± 0.28	25.68 ± 1.59	40.33 ± 0.37	−7.11 ± 0.96	40.96 ± 0.53	−9.99 ± 0.36	
32	10.44 ± 0.03	26.84 ± 2.60	38.46 ± 1.27	−6.28 ± 0.36	38.97 ± 0.99	−9.28 ± 0.63	
33	9.82 ± 0.25	28.09 ± 2.43	37.37 ± 1.15	−6.01 ± 0.32	37.85 ± 0.90	−9.15 ± 0.72	
34	9.51 ± 0.10	27.38 ± 2.14	37.44 ± 0.44	−5.65 ± 1.07	37.88 ± 0.54	−8.56 ± 1.25	
35	9.15 ± 0.20	27.68 ± 0.65	37.75 ± 1.07	−5.70 ± 0.93	38.18 ± 1.02	−8.57 ± 0.89	
36	9.89 ± 0.50	26.68 ± 0.31	40.12 ± 0.00	−7.25 ± 0.59	40.77 ± 0.18	−10.25 ± 0.74	
37	9.95 ± 0.51	27.08 ± 0.67	40.35 ± 0.31	−7.94 ± 0.15	41.12 ± 0.31	−11.13 ± 0.40	
38	9.98 ± 0.10	28.13 ± 1.27	38.35 ± 0.55	−6.85 ± 0.74	38.96 ± 0.59	−10.11 ± 0.76	
39	9.85 ± 0.32	27.99 ± 1.07	38.12 ± 2.19	−6.25 ± 0.30	38.63 ± 1.72	−9.31 ± 0.21	
40	9.71 ± 0.39	28.13 ± 0.19	37.95 ± 1.47	−6.00 ± 0.87	38.43 ± 1.25	−8.96 ± 0.77	
41	9.27 ± 0.31	27.53 ± 1.03	38.83 ± 0.08	−6.98 ± 0.41	39.46 ± 0.29	−10.19 ± 0.55	
42	8.85 ± 0.38	28.53 ± 0.51	37.87 ± 0.18	−6.53 ± 0.40	38.43 ± 0.31	−9.78 ± 0.55	
43	9.56 ± 0.13	27.51 ± 0.53	39.22 ± 0.59	−7.07 ± 0.31	39.85 ± 0.51	−10.22 ± 0.53	
44	9.48 ± 0.72	27.36 ± 2.37	38.96 ± 1.77	−6.49 ± 0.91	39.50 ± 1.50	−9.44 ± 0.73	
45	9.23 ± 0.50	27.26 ± 0.55	39.14 ± 2.21	−6.49 ± 0.78	39.68 ± 1.81	−9.40 ± 0.59	
46	9.79 ± 0.60	39.82 ± 1.09	25.81 ± 0.06	−7.85 ± 0.04	26.98 ± 0.56	−16.93 ± 0.50	
47	9.98 ± 0.26	39.78 ± 0.82	25.28 ± 0.46	−7.62 ± 0.10	26.40 ± 0.50	−16.77 ± 0.12	
48	11.25 ± 2.25	36.97 ± 6.06	28.12 ± 5.77	−7.12 ± 0.57	29.02 ± 4.45	−14.39 ± 1.36	
49	9.89 ± 0.64	40.19 ± 0.90	24.37 ± 0.13	−7.13 ± 0.12	25.39 ± 0.21	−16.30 ± 0.28	
50	9.48 ± 0.02	41.02 ± 1.31	23.17 ± 0.16	−6.44 ± 0.08	24.05 ± 0.33	−15.52 ± 0.12	
51	12.55 ± 0.31	35.47 ± 0.24	31.29 ± 0.85	−9.10 ± 0.02	32.59 ± 0.70	−16.23 ± 0.36	
52	12.50 ± 0.32	34.28 ± 0.47	32.36 ± 1.60	−8.94 ± 0.25	33.58 ± 1.16	−15.47 ± 0.89	
53	9.96 ± 0.02	39.83 ± 0.18	24.79 ± 1.54	−7.13 ± 0.63	25.79 ± 1.33	−16.05 ± 0.34	
54	10.00 ± 0.00	40.60 ± 0.93	23.43 ± 0.00	−5.90 ± 0.25	24.16 ± 0.36	−14.13 ± 0.46	
55	9.50 ± 0.70	40.38 ± 0.08	24.18 ± 2.26	−6.46 ± 0.96	25.03 ± 1.91	−14.93 ± 0.61	
56	10.38 ± 0.05	38.89 ± 0.57	28.49 ± 0.55	−9.01 ± 0.10	29.88 ± 0.52	−17.55 ± 0.40	
57	10.27 ± 0.22	38.68 ± 1.49	27.37 ± 0.52	−8.73 ± 0.38	28.73 ± 0.57	−17.69 ± 0.32	
58	10.39 ± 0.30	38.93 ± 1.33	26.17 ± 0.46	−7.59 ± 0.68	27.25 ± 0.63	−16.15 ± 0.86	
59	9.43 ± 0.22	41.35 ± 3.14	22.38 ± 3.00	−5.63 ± 1.40	23.08 ± 2.56	−13.98 ± 1.39	
60	11.13 ± 0.02	39.06 ± 1.07	26.50 ± 0.49	−8.13 ± 0.08	27.72 ± 0.50	−17.05 ± 0.22	

TAC: total anthocyanin content; cya-3-glu: cyanidin-3-*O*-glucoside; L*: luminosity; a*: red–green; b*: yellow–blue; C*: chroma; h: hue; RGB: color obtained with re-green-blue model.

**Table 4:** Individual anthocyanin identification and quantification of *Berberis vulgaris* wild freeze-dried fruit fraction (BVFF) and optimized colorant extract (BVE).

Peak	Rt	UV	[M]^+^	MS^2^	Tentative Identification	Quantification (mg/g, dw)	
						BVFF	BVE
1 ^A^	5.58	522	465	303(100)	Delphinidin-3-*O*-glucoside	3.54 ± 0.01 ^b^	5.27 ± 0.02 ^a^
2 ^B^	7.60	516	449	287 (100)	Cyanidin-3-*O*-glucoside	2.69 ± 0.004 ^b^	4.00 ± 0.01 ^a^
3 ^C^	8.98	524	479	317 (100)	Petunidin-3-*O*-glucoside	1.48 ± 0.001 ^b^	2.20 ± 0.002 ^a^
4 ^D^	10.35	507	433	271 (100)	Pelargonidin-3-*O*-glucoside	0.67 ± 0.001 ^b^	1.00 ± 0.002 ^a^
5 ^E^	14.05	526	493	331 (100)	Malvidin-3-*O*-glucoside	3.01 ± 0.004 ^b^	4.47 ± 0.01 ^a^

In the peak column, each capital superscript letter means the calibration curve used for quantification. A—delphinidin-3-*O*-glucoside (y = 1047.4x + 8.3518; R^2^: 0.99995); B—cyanidin-3-*O*-glucoside (y = 1217.5x + 3.2479; R^2^: 0.99996); C—petunidin-3-*O*-glucoside (y = 832.74x + 2.6594; R^2^: 0.99994); D—pelargonidin-3-*O*-glucoside (y = 1371.9x + 7.8528; R^2^: 0.99997); E—malvidin-3-*O*-glucoside (y = 1011x + 6.8629; R^2^: 0.99996). Different small superscript letters in each line mean statistically significant differences (*p* < 0.05) compared by Student’s *t* test.

**Table 5:** Chemical composition and bioactive properties of *Berberis vulgaris* freeze-dried fruit fraction (BVFF) and its optimized colorant extract (BVE).

CHEMICAL COMPOSITION										
Phenolic compounds										
	Total polyphenols (mg GAE/g, dw)		Hydroxybenzoic acids (mg GAE/g, dw)			Hydroxycinnamic acids (mg FAE/g, dw)			Flavonols (mg QE/g, dw)	
BVFF	159.90 ± 12.45 ^b^		47.30 ± 4.24 ^b^			31.09 ± 1.11 ^b^			7.41 ± 0.65 ^b^	
BVE	290.72 ± 22.64 ^a^		81.74 ± 0.76 ^a^			46.52 ± 4.13 ^a^			10.00 ± 0.73 ^a^	
BIOACTIVE PROPERTIES										
Antioxidant activity										
	in vitro chemical					in vitro biological				
	DPPH (mg TE/g, dw)		Folin–Ciocalteu (mg GAE/g, dw)			OxHLIA (IC_50_ values, µg/mL)				
BVFF	76.30 ± 4.00 ^b^		60.99 ± 3.83 ^b^			60 ± 3 ^b^				
BVE	111.37 ± 9.65 ^a^		88.03 ± 7.84 ^a^			125 ± 4 ^a^				
Antibacterial activity (mg/mL hydroethanolic extract)										
	BVFF		BVE		Streptomycin		Methicillin		Ampicillin	
	MIC	MBC	MIC	MBC	MIC	MBC	MIC	MBC	MIC	MBC
Gram-negative bacteria										
*Enterobacter Cloacae*	>10	>10	5	10	0.007	0.007	n.t.	n.t	0.15	0.15
*Escherichia coli*	5	>10	2.5	10	0.01	0.01	n.t.	n.t.	0.15	0.15
*Pseudomonas aeruginosa*	10	>10	5	10	0.06	0.06	n.t.	n.t.	0.63	0.63
*Salmonella enterica*	2.5	>10	1.25	20	0.007	0.007	n.t.	n.t.	0.15	0.15
*Yersinia enterocolitica*	>10	>10	10	20	0.007	0.007	n.t.	n.t.	0.15	0.15
Gram-positive bacteria										
	MIC	MBC	MIC	MBC	MIC	MBC	MIC	MBC	MIC	MBC
*Bacillus cereus*	10	>10	1.25	10	0.007	0.007	n.t.	n.t.	n.t.	n.t.
*Listeria monocytogenes*	10	>10	5	>20	0.007	0.007	n.t.	n.t.	0.15	0.15
*Staphylococcus aureus*	10	>10	2.5	20	0.007	0.007	0.007	0.007	0.15	0.15
Antifungal activity (mg/mL hydroethanolic extract)										
	BVFF		BVE		Ketoconazole					
	MIC	MFC	MIC	MFC	MIC	MFC				
*Aspergillus brasiliensis*	2.5	>10	5	>10	0.06	0.125				
*Aspergillus fumigatus*	10	>10	10	>10	0.5	1.0				

GAE: gallic acid equivalent; FAE: ferulic acid equivalent, QE: quercetin equivalent, TE: Trolox equivalent; IC_50_: extract concentration providing 50% of antioxidant activity. Different small superscript letters in each column mean statistically significant differences (*p* < 0.05) compared by Student’s *t* test. MIC: minimum inhibitory concentration; MBC: minimum bactericidal concentration; MFC: minimum fungicidal concentration. IC_50_ for Trolox OxHLIA essay = 21.8 ± 0.3 µg/mL.

## Data Availability

The original contributions presented in the study are included in the article/Supplementary Material, further inquiries can be directed to the corresponding author.

## Supplementary Materials

The following supporting information can be downloaded at: https://www.mdpi.com/article/10.3390/foods14020183/s1, Table S1. Estimated values, standard errors, *t*-statistics, *p*-values, and confidence intervals for each parameter in the model described by Equation (4).
