# Supplementary material for: Natural Food Colorant Obtained from Wild Berberis vulgaris L. by Ultrasound-Assisted Extraction: Optimization and Characterization
Source: Foods. 2025 Jan 9;14(2):183. doi: 10.3390/foods14020183 (PMC11764707; doi:10.3390/foods14020183)
Supplement: Supplementary file 1 [file foods-14-00183-s001.zip › foods-3394100-supplementary.pdf]

Table S1. Estimated values, standard errors, t-statistics, p-values, and confidence intervals for each parameter in the model described by Eq. 4

Parameter	Value	Standard Error	T-Statistic	P-Value	Confidence Interval
b_0	865,616	1,46E+00	592,002	3,27E-89	{862.671, 868.561}
b_1	67,0892	2,80743	23,897	3,31E-27	{61.4347, 72.7437}
b_2	3,62955	4,85E+00	0,748168	0,458251	{-6.14138, 13.4005}
b_3	-16,9832	9,69691	-1,75141	0,0866897	{-36.5138, 2.54735}
b_4	9,91258	1,62E+01	0,612242	0,543459	{-22.697, 42.5222}
b_{11}	-2,89838	3,28892	-0,881254	0,382863	{-9.52261, 3.72585}
b_{22}	-0,030752	3,00E-02	-1,02352	0,311533	{-0.0912664, 0.0297624}
b_{33}	0,106308	0,272305	0,3904	0,698083	{-0.442142, 0.654757}
b_{44}	-0,208484	0,368066	-0,56643	0,573916	{-0.949806, 0.532839}
b_{12}	-0,297198	0,273895	-1,08508	0,283664	{-0.848851, 0.254454}
b_{13}	-0,424065	0,878946	-0,48247	0,631811	{-2.19435, 1.34622}
b_{14}	-1,76301	0,674012	-2,61569	0,0120769	{-3.12054, -0.405477}
b_{23}	0,0929015	0,0641628	1,4479	0,154576	{-0.036329, 0.222132}
b_{24}	0,0584702	0,0492027	1,18835	0,240928	{-0.040629, 0.157569}
b_{34}	0,218318	0,157895	1,38269	0,173585	{-0.0996975, 0.536334}
